# Predictive ability of a process‐based versus a correlative species distribution model

**DOI:** 10.1002/ece3.6712

**Published:** 2020-10-08

**Authors:** Steven I. Higgins, Matthew J. Larcombe, Nicholas J. Beeton, Timo Conradi, Henning Nottebrock

**Affiliations:** ^1^ Plant Ecology University of Bayreuth Bayreuth Germany; ^2^ Department of Botany University of Otago Dunedin New Zealand; ^3^ CSIRO Hobart Tas. Australia; ^4^ School of Biological Sciences University of Tasmania Hobart Tas. Australia

**Keywords:** ecological niche model, extrapolation, invasive species, MaxEnt, mechanistic models, model transferability, TTR‐SDM

## Abstract

Species distribution modeling is a widely used tool in many branches of ecology and evolution. Evaluations of the transferability of species distribution models—their ability to predict the distribution of species in independent data domains—are, however, rare. In this study, we contrast the transferability of a process‐based and a correlative species distribution model. Our case study uses 664 Australian eucalypt and acacia species. We estimate models for these species using data from their native Australia and then assess whether these models can predict the adventive range of these species. We find that the correlative model—MaxEnt—has a superior ability to describe the data in the training data domain (Australia) and that the process‐based model—TTR‐SDM—has a superior ability to predict the distribution of the study species outside of Australia. The implication of this analysis, that process‐based models may be more appropriate than correlative models when making projections outside of the domain of the training data, needs to be tested in other case studies.

## Graphical abstract

Species distribution modeling is a widely used tool in many branches of ecology and evolution. However, evaluations of the transferability of species distribution models—their ability to predict the distribution of species in independent data domains—are rare. In this study, we show that process‐based models may have better transferability than correlative models.

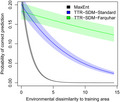

## 1 INTRODUCTION

Species distribution modeling (SDM) is a widely used tool in ecology, biogeography, invasion biology, and conservation biology. The role of SDMs in such studies varies from interpolating current species distributions, projecting past or future species distributions, to understanding how environmental resources and conditions control species' distribution (Guisan & Thuiller, 2005; Higgins, O'Hara, & Rӧmermann, 2012; Hijmans & Grahan, 2006; Peterson et al., 2011).

Climate change is shifting the ranges of species (Parmesan & Yohe, 2003; Steinbauer et al., 2018), which means that we are increasingly interested in the ability of SDMs to predict the potential future distribution of species. Similarly, we are interested in predicting the potential range of species outside of their native ranges. Assessments of how well SDMs predict distributions outside of the spatial or temporal domain of the training data are rare. For example, Guillera‐Arroita et al. (2015) report that only a handful of studies evaluate the ability of SDM models to predict independent data. Similarly, Hao, Elith, Guillera‐Arroita, and Lahoz‐Monfort (2019), in a review of the usage of the BIOMOD SDM software (Thuiller, Lafourcade, Engler, & Araújo, 2009), report that "the use of independent data to validate model performance is particularly uncommon."

Shabani, Kumar, and Ahmadi (2016) examined the ability of six different SDMs to predict the distribution of eight plant species. In this study, models were estimated using the global distribution of the species minus Australia and then used to predict their distribution in Australia. All species were non‐native in Australia. Magarey et al. (2018) examined the ability of four SDMs to predict the range of six species invasive to the USA. Distribution from the native ranges was used to estimate models and the observed distribution in the USA was used to assess the models. Both studies included both correlative and process‐based SDMs. Neither study reached a clear conclusion. Shabani et al. (2016) found no model consistently performed best and therefore advocated that users adopt an ensemble approach. Magarey et al. (2018) also declined to declare a winner, due to uncertainty about the quality of the data used for calibrating and testing the models.

To understand the performance of SDMs, we need to consider their underlying philosophies. SDMs span a continuum from correlative to mechanistic models (Dormann et al., 2012). Purely correlative models predict distributions by describing relationships between geographical occurrence and environmental data without explicit consideration of ecological processes. Fully mechanistic models (also called process‐based models), by contrast, predict distributions using mathematical functions of ecological processes (e.g., physiology, demography, and dispersal). Correlative models are by far the most widely used, and because they use distribution data they implicitly include the effects of biotic interactions and dispersal limitations on distribution (Sillero, 2011). On the other hand, fully processes‐based models do not capture dispersal and biotic interactions in this way. This implies that correlative models describe something closer to the realized niche while process‐based models describe something closer to the fundamental niche.

A model's position along the correlative to processes‐based continuum (Dormann et al., 2012) may determine its applicability to certain tasks. Correlative models may be useful for inferring distributions within the range of cryptic species, but biotic interactions, dispersal limitations, and persistence in unsuitable environments (Pagel et al., 2020) may limit their ability to extrapolate (Sillero, 2011). Process models, in contrast, are less flexible in describing the relationships between the environment and distribution and they typically ignore biotic interactions, which may limit their performance within a species' native range. However, their focus on mechanisms may allow them to make better projections outside of the native range (Elith, Kearney, & Phillips, 2010; Kearney & Porter, 2009).

The aim of this study is to address the question—can species distribution models predict independent species distribution data? The case study involves two taxonomic groups, the Australian acacias and the Australian eucalypts, and two models, MaxEnt (Phillips, Anderson, & Schapire, 2006) and the TTR‐SDM (Higgins et al., 2012). The acacia and eucalypt system has several advantages (Higgins & Richardson, 2014). First, there are a relatively large number of species in these groups in Australia, and the Australian Virtual Herbarium (AVH, www.avh.chah.org.au) data on their distribution is of high quality. Second, several species in these groups have been introduced outside of Australia, some of which have become naturalized, while others have become invasive (Richardson & Rejmánek, 2011). The distributions of the invasive and naturalized species are sampled by the GBIF (the Global Biodiversity Information Facility; www.gbif.org) record, thereby providing us with an independent, but methodologically equivalent, data set of the distribution of these species outside of their native range.

The two species distribution models considered in this study represent a correlative species distribution model (MaxEnt, Phillips et al., 2006) and a species distribution model that is based on a process model of plant growth (TTR‐SDM, Higgins et al., 2012). Both models use georeferenced environmental and species occurrence data to estimate the suitability of the environment at a geographic location for a species. The correlative model, MaxEnt, uses techniques from machine learning and multiple regression to explain the observed species distribution data. The model is well established in the literature and widely used by practitioners (Elith et al., 2011). Guillera‐Arroita et al. (2015) for instance report that more than 50% of SDM studies use MaxEnt. MaxEnt is a correlative model because it seeks statistical explanations of patterns observed in the data, it does not specify the biological or ecological processes that underlie these patterns. The TTR‐SDM (Higgins et al., 2012) by contrast is process‐based because it proposes that a physiological model of plant growth can explain the observed distribution of a species.

Our first hypothesis is that the correlative model, MaxEnt, will provide better fits to the Australian distribution data (the training data) because it is flexible and effective in finding functional forms that describe the relationship between the environmental and distributional data. Our second hypothesis is that the process model, TTR‐SDM, will provide better predictions of the outside of Australia distribution data (the independent testing data). These hypotheses are in line with previous work (Evans, Merow, Record, McMahon, & Enquist, 2016; Higgins et al., 2012; Kearney & Porter, 2009) and motivated by the expectation that a process‐based model, that adequately represents the processes controlling distribution, should transfer beyond the training domain.

## 2 METHODS

### 2.1 Study species, distribution and environmental data

The starting point for species selection was the list of Australian acacias and eucalypts. From these lists, we selected the 384 acacia and 374 eucalypt species that have been introduced outside of Australia (Higgins & Richardson, 2014). Distribution data on species within Australia were downloaded from the Australian Virtual Herbarium (AVH). The AVH data are probably the highest quality source of plant distribution data (see Daru et al., 2018 for a discussion of bias in the AVH and other similar data sets). For this same set of species, we downloaded species records outside of Australia from GBIF using the R package *rgbif* (Chamberlain et al., 2019).

The environmental data used for fitting the TTR‐SDM are defined in Higgins et al. (2012). The model uses minimum, mean and maximum monthly temperatures (from Hijmans, Cameron, Parra, Jones, & Jarvis, 2005), monthly soil moisture contents (from Trabucco & Zomer, 2010), solar radiation (from Trabucco & Zomer, 2010), and soil nitrogen content (from Shangguan, Dai, Duan, Liu, & Yuan, 2014). This exact same list of environmental variables was used for the MaxEnt model fitting. This ensures that both models use the same information for estimating the species distribution models. All environmental variables are available globally at an approximate spatial resolution of 1 km × 1 km.

How one samples pseudoabsence points are influential (Aarts, Fieberg, Brasseur, & Matthiopoulos, 2013), even if other decisions made in species distribution modeling can be more influential (Barbet‐Massin, Jiguet, Albert, & Thuiller, 2012). To sample pseudoabsence points, the environmental data described in the previous paragraph were classified using the *clara* algorithm (R package *cluster*, Maechler, Rousseeuw, Struyf, Hubert, & Hornik, 2019) into 24 environmental zones. The environmental zone classification is shown in Figure S1. A maximum of 1,000 presence data points per species were sampled, and these presence points were sampled evenly across environmental zones. We sampled the same number of pseudoabsence points as presence points (the actual number varied slightly due to integer rounding). Half of the pseudoabsence points were sampled from within Australia and sampling was stratified by the environmental zones in Australia, the other half of the pseudoabsence points were sampled from outside of Australia and these samples were stratified by the 24 environmental zones that occur globally. This strategy was designed to ensure that a broad range of environments—including environmental types that do not occur in Australia—were included in the pseudoabsence sample. Exactly, the same presence and absence data were used by MaxEnt and TTR‐SDM.

### 2.2 The inverse method of parameter estimation used by the TTR‐SDM

For many problems in ecological forecasting process‐based models are used to predict the state variables of ecological systems. For example, let us assume we wish to predict the distribution of a species in geographic space, and let us assume we have a model capable of making such predictions (e.g., the TTR‐SDM). The model parameters can either be estimated using forward or inverse methods; sometimes these two approaches are called direct and indirect methods. In the case of the forward method, we use direct observations to estimate the model's parameters. For example, assuming the model includes parameters that describe the temperature dependency of biomass growth, one could estimate these by conducting an experiment that creates observations of plant growth along an experimental temperature gradient. In the case of the inverse method, we might use observations of a species' distribution along a temperature gradient to indirectly estimate the temperature dependency of plant growth. That is, the forward method predicts the distribution of the species using direct estimates of the parameters that describe the temperature dependency of growth, whereas the inverse method predicts the parameters that describe the temperature dependency of growth using observations of the species' distribution. Hence process‐based models can either use forward or inverse parameterization and the same process‐based model can use either forward or inverse parameterization or a combination of the two.

Most applications of process‐based species distribution models use forward parameterization methods (Chuine, 2000; Enriquez‐Urzelai, Kearney, Nicieza, & Tingley, 2019; Hackett & Vanclay, 1998; Kearney & Porter, 2009; Sutherst, 2003; Sykes, Prentice, & Cramer, 1996). Forward species distribution models have the attractive feature that they use independent information on lower‐level processes (e.g., physiology) to predict a higher‐level phenomenon (e.g., distribution). Furthermore, direct parameter estimation using experimental assays eliminates the possibility of misspecifying the parameterization. The disadvantage is that the parameterization process requires high effort, which in turn prohibits the use of such approaches for large suites of species. A more comprehensive review of developments in process‐based species distribution modeling and the promise of inverse methods is provided by Evans et al. (2016).

In this study, we use the TTR‐SDM (Higgins et al., 2012), which is a process‐based species distribution model, and we estimate the parameters of this model using inverse methods. The TTR‐SDM is based on the Thornley Transport Resistance model (Thornley, 1998) but expands the Thornley model to explicitly consider how variation in environmental conditions influences plant growth. We use the model to predict plant biomass, conditional on the environmental covariates listed in the previous section. That is, the model simulates plant growth at geographic point locations forced by monthly variation in environmental covariates. Specifically, the model's nitrogen uptake, carbon assimilation (photosynthesis), and growth parameters are assumed to be influenced by monthly variation in temperature, soil moisture, solar radiation, and soil nitrogen (the latter is assumed to be constant over all months). Carbon gain is assumed to have a trapezoidal dependency on temperature and saturating dependencies on solar radiation and soil moisture. Each of these factors colimit as prescribed by Leibig's law of the minimum. That is, the model can mimic, for example, that temperature may limit in winter, but soil moisture in summer. Similarly, nitrogen uptake by roots is colimited by a trapezoidal dependency on soil moisture, and saturating dependencies on soil nitrogen and soil temperature. Growth itself is a trapezoidal function of temperature and respiration is a saturating function of temperature. The inverse parameter estimation task is to estimate the parameters of these trapezoidal and saturating functions. The biological justification for these assumptions and the equations used are provided in Higgins et al. (2012).

We run the model forward until biomass reaches a steady state. This biomass estimate is then transformed using the complementary log–log function to the probability of the species being present. The likelihood of observing the data given this probability is assumed to follow the Bernoulli distribution (see Higgins et al., 2012 for further details). We estimate the parameters using stochastic simulation methods that identify parameter combinations that produce range projections consistent with the observed data. Although a diversity of algorithms exist, we have found that the differential evolution genetic algorithm (Mullen, Ardia, Gil, Windover, & Cline, 2011; Price, Storn, & Lampinen, 2006) to be efficient and reliable for the inverse estimation the TTR‐SDM's parameters.

Implicit in this discussion is that the parameter estimates are constrained by the model's structure. That is, how an environmental factor influences the projected species distribution is constrained by the model's assumptions. For example, the TTR‐SDM assumes that carbon uptake is a saturating function of soil moisture, but a unimodal function of daytime air temperature (equations 17 and 15 in Higgins et al., 2012). This makes the model less flexible than correlative models because the model defines a priori which environmental factors influence which physiological processes, and the functional form of this influence.

### 2.3 Fitting the species distribution models

For any species with more than 15 observation points (presence in at least 15 grid cells for which all environmental data is available), a model fit was attempted. The 25%, 50%, and 75% quantiles of the number of presence points used in the models were 51, 98, and 191.

For MaxEnt, we use the *maxnet* function (and its default settings) of the R package *maxnet* (Phillips, 2017). For the TTR‐SDM, we use two variations. The first is exactly as described by Higgins et al. (2012); the second involves the replacement of the net photosynthesis function (see equation 14 in Higgins et al., 2012) with a Farquhar style photosynthesis function (see Conradi et al., 2020 for a case study that uses this model variant). This means that the parameters associated with this function (labeled β_1_ − β_8_ in Higgins et al., 2012) are no longer estimated from the distribution data. Rather, these environmental effects on photosynthesis are predefined by the Farquhar photosynthesis model (Farquhar, von Caemmerer, & Berry, 1980). For the photosynthesis model, we use the C3 photosynthesis model as described by von Caemmerer (2000). Although the parameters of the Farquhar model vary with species, we simplify this analysis by assuming that they are universally valid for all species in this study. The TTR‐Farquhar‐SDM thus uses 8 less free parameters than the TTR‐Standard‐SDM (21 vs. 29 parameters). The Supporting Information provides a listing of the Farquhar model equations used and the parameter values used. The Farquhar model variation further requires an estimate of the atmospheric CO_2_ concentration which we assumed was 338 ppm (the approximate concentration in 1980).

### 2.4 Model assessment

We evaluated the model fits using a range of standard procedures based on the confusion matrix (the matrix containing the number of true‐positive TP, false‐positive FP, true‐negative TN, and false‐negative FN classifications). Sensitivity (*S*) is the proportion of true positives *S* = TP/(TP + FN), it ranges between 0 and 1 with *S* = 1 implying the model perfectly predicts all observations. Specificity (*s*) is the proportion of true negatives *s* = TN/(FP + TN). Prevalence *P* is the proportion of the modeled domain predicted to be suitable, *P* = (TP + FP)/(TP + TN + FP + FN); high prevalence models would have a perfect sensitivity but would be useless. Bias was calculated as *B* = *P* − (TP + FN)/(TP + TN + FP + FN) where positive values indicates over‐prediction and negative values under‐prediction. We further calculate the TSS true skills statistic (Allouche, Tsoar, & Kadmon, 2006) defined as TTS = *S* − *s*.

The TSS is a threshold dependent measure of model performance, which is preferred in situations where the ability to predict presences and absences is assessed (Allouche et al., 2006). TSS behaves similarly to the widely used AUC. The AUC or area under the receiver operator curve (the line describing how the true‐positive rate increases with the true‐negative rate) can be used to assess the ability of a species distribution model to classifying locations into two classes—presence and absence locations. An AUC value of 1.0 means that the model perfectly classifies the presence and absence records, whereas an AUC of 0.5 means that the model has no ability to classify presence and absence locations (it is no better than random). An AUC value of 0.9 implies that there is a 90% chance that the model can separate presence and absence records. Some authors (Lobo, Jiménez‐Valverde, & Real, 2008) argue that the AUC is not an objective measure of model performance when there are no true absences; however, the AUC values of different models fitted to the same data are informative. AUC further avoids the user having to make decisions on how to split the environmental suitability metrics produced by SDMs into presence and absence categories.

## 3 RESULTS

**Figure 1:**
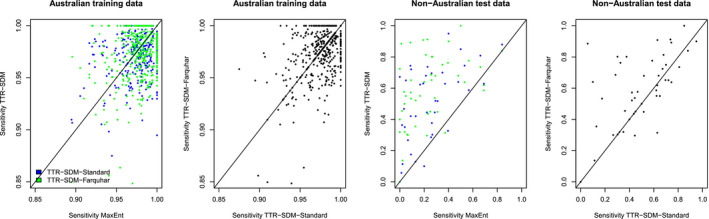
Pairwise comparison of the sensitivity of MaxEnt versus TTR‐SDM models and for the standard version of the TTR‐SDM versus the Farquhar version of the TTR‐SDM. Australian training data indicate statistics calculated within the training region. Non‐Australian test data indicate statistics for predictions made outside of Australia evaluated against GBIF records outside of Australia. Each data point represents a single species (*n* = 664 for the training data, *n* = 46 for the test data)

For all models, we used a threshold that maximized the true presences and true pseudoabsences to transform the modeled suitability [0..1] into presence‐absence maps [0,1], for which we calculated the confusion matrices and associated metrics. MaxEnt and TTR‐SDM had similar sensitivities when calculated using the training data (Figure 1). We similarly did not detect any systematic differences in the sensitivities of the two variants (without and with Farquhar photosynthesis) of the TTR‐SDM when using the training data. The test data set of observations of the species outside of their native Australia was restricted to species with presences in at least 20 10 × 10 km grid cells (46 species). For this test data set, the sensitivity of the TTR‐SDM was higher than that of MaxEnt (mean difference of 0.25 and 0.35 for the TTR‐SDM‐Standard and TTR‐SDM‐Farquhar, respectively; both differences *p *< .0001 with a paired *t* test). The TTR‐SDM‐Farquhar tended to have higher sensitivities (32 of 46 species) than the TTR‐SDM‐Standard (for similar plots of the specificity metrics see Figure S2).

**Figure 2:**
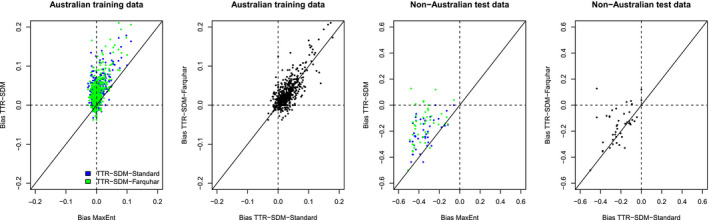
Pairwise comparison of the bias of MaxEnt versus TTR‐SDM models and for the standard version of the TTR‐SDM versus the Farquhar version of the TTR‐SDM. Australian training data indicate statistics calculated within the training region. Non‐Australian test data indicate statistics for predictions made outside of Australia evaluated against GBIF records outside of Australia. Each data point represents a single species (*n* = 664 for the training data, *n* = 46 for the test data)

The bias statistics revealed that for the training data MaxEnt had low bias and this bias was more symmetrical around zero than for the TTR‐SDM (Figure 2). The TTR‐SDM had a positive bias (a tendency to over‐predict prevalence). For the training data, there were no clear differences in the bias of the two variants of the TTR‐SDM. For the test data, MaxEnt's bias was always negative (a tendency to under‐predict) and for the TTR‐SDM the bias was mostly negative (negative for 43 and 39 of 46 species, respectively, for TTR‐SDM‐Standard and for TTR‐SDM‐Farquhar). The bias for the test data was barring a few species closer to zero for the TTR‐SDM than for MaxEnt, indicating that the prevalence error (level of under‐prediction) was systematically more severe when using MaxEnt (mean difference of 0.14 and 0.20 for the TTR‐SDM‐Standard and TTR‐SDM‐Farquhar, respectively; both differences *p* < .0001 with a paired *t* test). The TTR‐SDM‐Standard model tended to stronger under‐prediction than the TTR‐SDM‐Farquhar.

**Figure 3:**
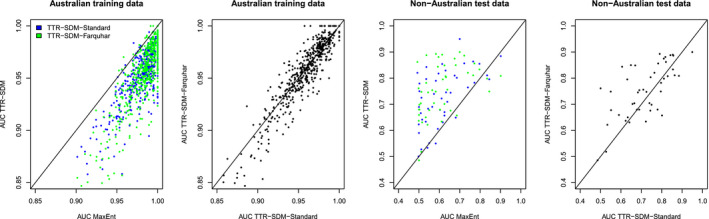
Pairwise comparison of the AUC statistics of MaxEnt versus TTR‐SDM models and for the standard version of the TTR‐SDM versus the Farquhar version of the TTR‐SDM. Australian training data indicate statistics calculated within the training region. Non‐Australian test data indicate statistics for predictions made outside of Australia evaluated against GBIF records outside of Australia. Each data point represents a single species (*n* = 664 for the training data, *n* = 46 for the test data)

The AUC metrics revealed that MaxEnt models were mostly better at predicting the training data than the TTR‐SDMs (Figure 3). There were no clear systematic differences in the AUC statistics for the training data for the two TTR‐SDM model variants. When AUC was calculated from the test data, the values were higher for the TTR‐SDM than for the MaxEnt models (mean difference of 0.11 and 0.14 for the TTR‐SDM‐Standard and TTR‐SDM‐Farquhar, respectively; both differences *p* < .0001 with a paired *t* test). The AUC values for the two variants of the TTR‐SDM were similar. The TSS was highly correlated with the AUC metrics (see Figure S3); we therefore do not discuss the TSS further here.

**Figure 4:**
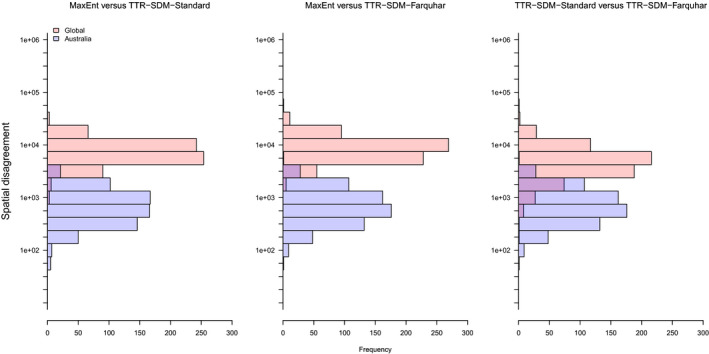
Frequency distributions of the spatial disagreement between the projections of models for 664 species of Australian acacias and eucalypts, calculated as the sum of the absolute differences in the presence‐absence predictions for 0.25 degree grid cells. The global distributions represent the disagreement observed globally, the Australian distributions represent disagreement observed in Australia

The models produced not only fits of different quality when assessed using TSS and AUC, they also differed considerably in the areas predicted to be suitable. We calculated the spatial disagreement between models as the sum of the number of quarter degree cells that disagree in their presence/absence classification (Figure 4). The area of a quarter degree cell varies with latitude, but as an approximate reference: A spatial disagreement of 1,000 quarter degree cells is about the size of Mexico and a spatial disagreement of 100 is slightly larger than the United Kingdom. The largest differences in the projections were between MaxEnt and both TTR‐SDM variants and these differences were typically 10,000 quarter degree cells. The differences between the two TTR‐SDM variants were slightly smaller, but still substantial for many species. The disagreements within the training area (Australia) were by definition smaller. The disagreement was larger between MaxEnt and the two TTR‐SDM variants than between the two TTR‐SDM variants. There was, however, a high variance in the disagreement between the two TTR‐SDM variants in Australia.

**Figure 5:**
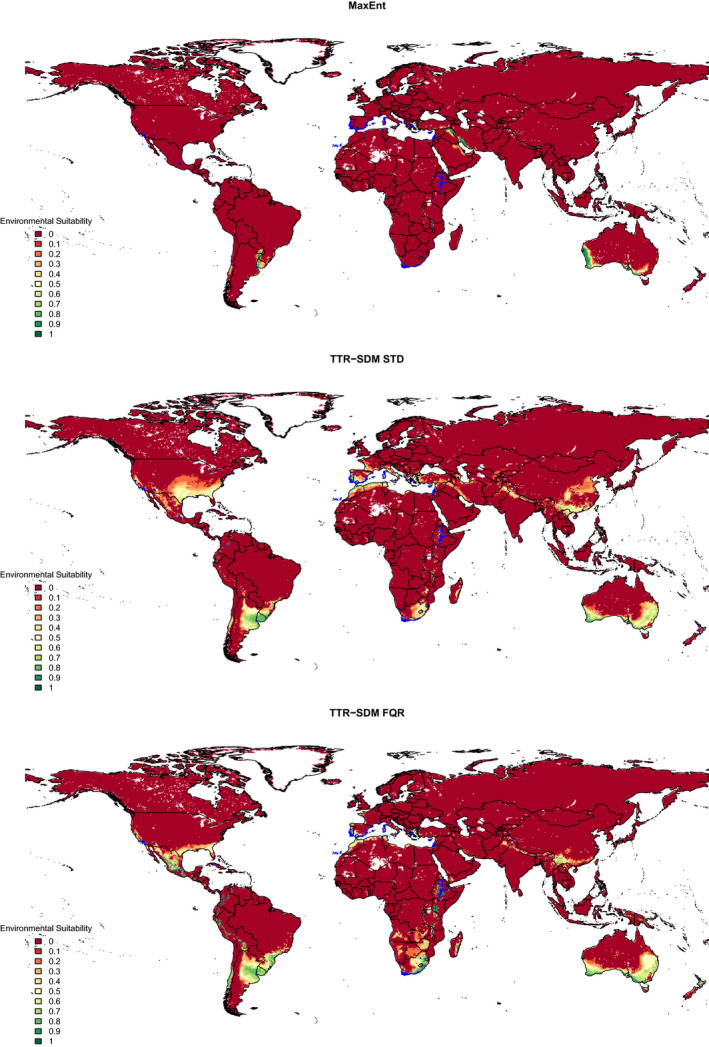
Global projected environmental suitability of *Acacia saligna* for three different species distribution models. The species distribution models were trained using data from Australia. The projections are made for 0.25 degree grid cells. Blue crosses indicate GBIF records outside of Australia

To visualize the disagreements between model projections, we plot the projected environmental suitability surfaces for the three models for *Acacia saligna* (Figure 5). This illustrates the more positive bias of both TTR‐SDM variants reported in Figure 2. In this example, the TTR‐SDM‐Farquhar correctly predicted 262 of 322 adventive records outside of Australia (presences in 10 × 10 km grid cells), whereas TTR‐SDM‐Standard and MaxEnt correctly predicted 219 and 69 of these records. MaxEnt predicts the occurrences in the Western Cape, South Africa, but misses occurrences in Ethiopia, Mexico, California, and on the Iberian Peninsula. The TTR‐SDM‐Standard and TTR‐SDM‐Farquhar predict suitability in all of these regions, but the TTR‐SDM‐Farquhar was able to get more of the recorded occurrences correct.

**Figure 6:**
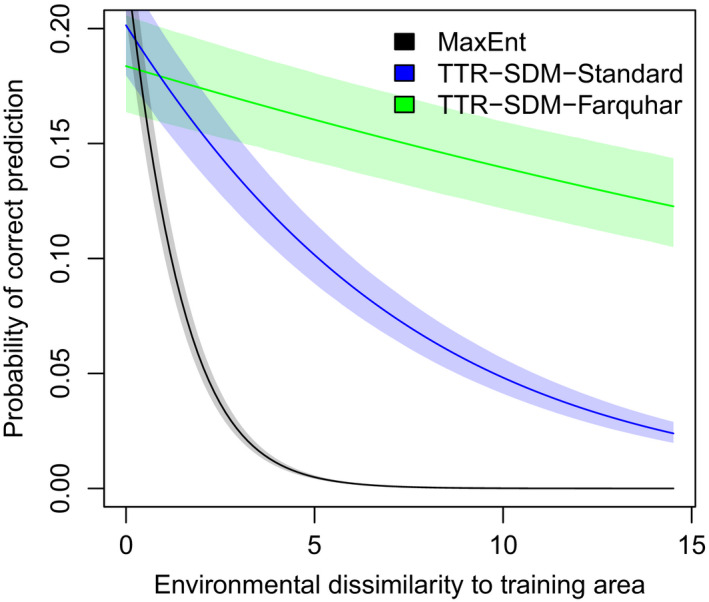
The probability of the species distribution models correctly predicting GBIF records of the study species outside of Australia. The lines represent the fits of a Bayesian logistic regression model to an environmental dissimilarity score (Figure S4). The dissimilarity score indicates dissimilarity relative to the training arena. The shaded areas indicate the 95% credible intervals.

Differences in the models performance in predicting GBIF records outside the training arena may be related to how dissimilar the test arena was to the training arena. To examine this possibility, we calculated a dissimilarity score (the Nt2 index described by Mesgaran, Cousens, & Webber, 2014). This index estimates how dissimilar environmental variates in the test domain are from those in the training domain. Nt2 scores close to zero indicate similarity (in terms of both univariate range and multivariate combination), whereas values that exceed 1 indicate data points outside of the domain defined by the training data (Mesgaran et al., 2014). Figure S4 illustrates this index for this study, where Australia defines the training domain. Using logistic regression, we find that the probability of correctly predicting a GBIF occurrence record (present in a 10 × 10 km grid cell) decreases with increasing dissimilarity. This decrease in predictive ability is steepest for MaxEnt, and shallowest for the TTR‐SDM‐Farquhar (Figure 6). The logistic regression model was fitted using Bayesian MCMC methods using JAGS (Plummer, 2003). The linear predictor of this model took the form, *y* = intercept[model] + slope[model] | species, that is species is treated as a random effect on the intercept and an intercept and slope are estimated for each SDM model.

## 4 DISCUSSION

Evaluations of the performance of species distributions mostly rely on partitioning a single data set into training and testing subsets (Hao et al., 2019), rather than evaluating performance in independent data domains, such as at a different point in time or in a different biogeographic region. This study used independent data domains for training and testing. The study also contrasts two different philosophies of species distribution modeling—namely correlative and process‐based species distribution modeling, even though we acknowledge that process and correlative models exist on a continuum (Dormann et al., 2012) and emphasize that process models can be parameterized using either forward or inverse methods.

Our results illustrate that the correlative model—MaxEnt—is excellent at creating distribution models that describe occurrence data of Australian eucalypt and acacia species derived from the Australian Virtual Herbarium. Although absolute values of AUC should not be compared between data sets when using pseudoabsence data, models with values of circa 0.7 are considered useful because they indicate a 70% chance that a random presence record will be ranked higher than a random absence record (Lobo et al., 2008). The lower quartile, median, and mean AUC values of the MaxEnt models in this study were 0.9794, 0.9923, and 0.9858 when evaluated against the training data. These same statistics for the process‐based model—TTR‐SDM—were lower, but also good for both the standard (0.9505, 0.9692, and 0.9646) and Farquhar (0.9482, 0.9709, and 0.9637) version of the TTR‐SDM. Our analyses also showed that MaxEnt performs with low and neutral bias on the training data, whereas the TTR‐SDM had, for the training data, higher bias and this bias was skewed toward over‐prediction.

When it comes to evaluating against independent data the TTR‐SDM performed better than MaxEnt. Here, the first quartile, median, and mean AUC values were 0.5201, 0.5943, and 0.6119 for MaxEnt, and 0.6432, 0.7324, 0.7195 and 0.6795, 0.7550, 0.7541 for TTR‐SDM‐Standard and TTR‐SDM‐Farquhar. The bias of the projections made with MaxEnt was negative, whereas for the TTR‐SDM models it was, although skewed toward negative values, either less biased than MaxEnt or slightly positively biased.

The better performance of the TTR‐SDM in predicting the distribution of species outside of the training data domain may be due to the process‐based nature of the model. The TTR‐SDM represents a series of explicit hypotheses of how environmental factors influence resource assimilation and growth of plants and this imposes a rigidity which makes over‐fitting less likely (Higgins et al., 2012). The premise is that these relationships between physiology and the environmental are universal and that the TTR‐SDM models should therefore transfer beyond the training data domain better than a purely correlative model such as MaxEnt. This is indeed what we see when we compare the probability of correctly predicting occurrence data outside of the training domain for MaxEnt, TTR‐SDM‐Standard, and TTR‐SDM‐Farquhar (Figure 6). This probability is similar for all three models for locations that are environmentally similar to the training data domain (the region of overlapping credible intervals in Figure 6 where environmental dissimilarity is <1), but for MaxEnt it declines rapidly with increasing dissimilarity. This decline is slower for both TTR‐SDM variants, but slowest for the TTR‐SDM‐Farquhar which includes more process detail than TTR‐SDM‐Standard.

It is tempting, but in our opinion misleading, to propose that a process‐based model such as the TTR‐SDM will describe the fundamental niche and a correlative SDM such as MaxEnt the realized niche. The TTR‐SDM articulates a hypothesis for the potential physiological niche of a plant species, which one could interpret as an approximation of the fundamental niche. It is, however, unlikely that this niche construct can be estimated using inverse methods from data on the realized distribution of species because such distribution patterns are influenced by not only the fundamental niche, but also by biotic interactions and dispersal processes. The TTR‐SDM would get closer to describing the fundamental niche if it used forward parametrization. Such a model would identify geographic locations that are ecologically suitable in the absence of biotic interaction (Wisz et al., 2013). We therefore suggest that the TTR‐SDM when using inverse parameterization (as in this study) falls toward the process end of the Dormann et al. (2012) continuum, but is some distance from the process end.

Overall, the predictive accuracy of all models in this study on independent data was rather poor. There are several possible explanations for this. First, the data although good for the training region (Australia) are poor outside of the training region. We estimated models for 664 species that have been introduced outside of Australia (Higgins & Richardson, 2014), yet only 46 of these species have GBIF records of presence in 20 or more 10 × 10 km grid cells outside of Australia. Furthermore, the records may be biased because in many regions outside of Australia the species are currently expanding their ranges and it is possible that in other parts of their non‐native range, their distribution is restricted by management actions (Richardson, Hui, Nuñez, & Pauchard, 2014; Richardson & Rejmánek, 2011).

A further explanation for the poor accuracy is that the models themselves are poor. For comparison, we review some prominent studies which have evaluated SDM models for plants in independent data domains. Crimmins, Dobrowski, and Mynsberge (2013) used an ensemble of models to predict observed range shifts of native species between circa 1930 and 2005. The models were trained on the 1930s data and tested using the 2005 data. Although they also found that the AUC values of the models were higher with the training data than with the test data, the declines in AUC they reported were smaller (circa 0.10) than the declines observed in the current study. The better performance could be attributed to the different models used in Crimmins et al. (2013), but it could also be due to the better quality of data for both model fitting and calibration. The Crimmins et al. (2013) study used presence‐absence data from plot surveys. In total, 1,376 plots were surveyed in the 1930 survey and 33,596 plots in the 2005 survey. On balance, the reported performance might have been better in Crimmins et al. (2013) because of the high‐quality test data set and due to the fact that the model transfer was within a biographic region. When transferring between biogeographic regions it is likely that species will be confronted with an entirely new set of biological interactions. In correlative and inversely fitted process‐based SDMs biological interactions are indirectly included in the parameter estimates; in a new biogeographic domain, this inadvertent parameterization of biotic interactions may have more severe impacts than within the same biogeographic realm. This problem is not necessary resolved by joint SDMs; joint SDMs can infer biotic interactions, but they can only infer the influence of biotic interactions observed in the training domain.

Other studies also suggest low transferability is common. For example, Randin et al. (2006) tested transferability of models trained in Austria and Switzerland by testing whether models developed using Austrian data could predict Swiss data (and vice versa). They found that the performance in the testing domain was poor. Similarly, Heikkinen, Marmion, and Luoto (2012) report low transferability in a study using a variety of organism types, including plants. Interestingly MaxEnt, which performed poorly relative to TTR‐SDM in this study, was in Heikkinen et al. (2012) one of the best, if not the best, performing model in both the training domain and in the test domain.

There are many decisions that need to be made in designing a model comparison. Our philosophy was to treat the two models as similarly as possible: same presence data, same pseudoabsence data, same environmental data, same training and test data domains, no cross‐validation in the training data domain. It is likely that different MaxEnt decisions (pseudoabsence sampling, regularization coefficients, feature selection, and clamping) would change the results we present here (Elith et al., 2011; Merow, Smith, & Silander, 2013). Such changes may well improve the performance of the MaxEnt models (Moreno‐Amat et al., 2015). In particular, it is likely that changing the regularization coefficients could reduce the over‐fitting of MaxEnt, which might improve its transferability. In this study, we provided both models with the same presence, pseudoscience and environmental data and use the default settings of the models. This favors the TTR‐SDM since it has rather precise data requirements and this study met those requirements. MaxEnt was, in contrast, forced to do the best it could with environmental data tailored for the TTR‐SDM. In addition, MaxEnt applications typically use larger number of pseudoabsence points than we used in this study (Renner et al., 2015)—using more pseudoabsence points might have improved the MaxEnt models in this study.

Hao et al. (2019)’s review of studies using BIOMOD (Thuiller et al., 2009) reports that only 3 of 109 studies using BIOMOD included an evaluation of transferability. In general, it would be helpful for SDM development to have a series of data sets for testing the transferability of species distribution models. This would make it easier for SDM model developers to evaluate the transferability. For example, it would be interesting to repeat our analyses on the Crimmins et al.(2013) data set. It would, furthermore, be valuable to expand such transferability tests to other process‐based models for plants. A range of promising physiological process models for species distributions models have been proposed (Chuine, 2000; Hackett & Vanclay, 1998; Higgins et al., 2012; Sutherst, 2003; Sykes et al., 1996), and there are also promising demographic process models of species distribution emerging (Merow et al., 2014; Schurr et al., 2012). Even complex ecosystem models such as Dynamic Global Vegetation Models can and have been interpreted as species distribution models (Cheaib et al., 2012).

In the introduction, we suggested that MaxEnt is the most widely used SDM. Another popular approach is the ensemble approach (in particular, the approach made accessible by BIOMOD, Thuiller et al., 2009). Several authors have concluded that ensembles are better than single models (Hao et al., 2019). We caution, however, that Crimmins et al. (2013) provides at least one exception and Elith et al. (2011) advised against ensemble approaches, arguing that our knowledge about the performance of different SDMs still needs to mature. Furthermore, if the majority of the models in an ensemble are based on similar assumptions then the ensemble prediction will be biased and we therefore echo Elith et al. (2011): understanding such biases through careful assessment of the ecological plausibility of models in well‐understood cases should be a priority.

In this study, we considered two variants of the process‐based TTR‐SDM. In the first, the rate of carbon assimilation is constrained by a series of trapezoidal functions that allow air temperature, solar radiation, moisture, and soil nitrogen to colimit carbon assimilation. The second, the rate of carbon assimilation is defined by a Farquhar‐type photosynthesis model. This ensured that the temperature, light, CO_2,_ and radiation dependency of photosynthesis are predefined by the Farquhar model and no longer estimated from the distribution data. This adds further rigidity to the model, which constrains the values other parameter values can assume. The Farquhar variant of TTR‐SDM performed similarly to the more flexible, standard variant, in the training domain, but was slightly less biased and had marginally better AUC values in the test domain. Moreover, the Farquhar variant performed better than the standard variant at sites that were dissimilar to the training data domain. That is including more physiological constraints into the model and estimating 8 less parameters from the distribution data did not lead to a clear reduction in the model's ability to describe the training data and improved the model's performance outside the testing domain.

In conclusion, the hypotheses posed in the introduction are supported by this case study: (a) the correlative model—MaxEnt—was superior to the process‐based model—TTR‐SDM—in describing the distribution data in the training domain; (b) the process‐based model was superior to the correlative model in predicting the distribution of plant species in the test domain.

## CONFLICT OF INTEREST

We declare no conflict of interest.

## AUTHOR CONTRIBUTION

**Steven I. Higgins:** Conceptualization (lead); Formal analysis (lead); Funding acquisition (lead); Writing‐original draft (lead); Writing‐review & editing (lead). **Matthew J. Larcombe:** Conceptualization (lead); Writing‐review & editing (equal). **Nicholas J. Beeton:** Conceptualization (lead); Writing‐review & editing (equal). **Timo Conradi:** Writing‐review & editing (supporting). **Henning Nottebrock:** Writing‐review & editing (supporting).

## Supporting information

Fig S1‐S4

Supplementary Material

## Data Availability

The data used in this study were downloaded from open access data bases www.gbif.org and www.avh.chah.org.au. The doi's of the downloads used in this study are acacia download https://doi.org/10.26197/5cf641a2df07c; eucalypt download https://doi.org/10.26197/5cf641c6aa20f.
