# Supplementary material for: Predictive ability of a process‐based versus a correlative species distribution model
Source: Ecol Evol. 2020 Oct 8;10(20):11043–54. doi: 10.1002/ece3.6712 (PMC7593166; doi:10.1002/ece3.6712)
Supplement: Supplementary file 1 — Fig S1‐S4 [file ECE3-10-11043-s001.pdf]

ORIGINAL ARTICLE

Supplementary Figures

Supplementary Figures for predictive ability of a process-based versus a correlative species distribution model

Steven I. Higgins[1*] | Matthew J. Larcombe[2] | Nicholas J. Beeton[3,4] | Timo Conradi[1] | Henning Nottebrock[1]

Supporting Information

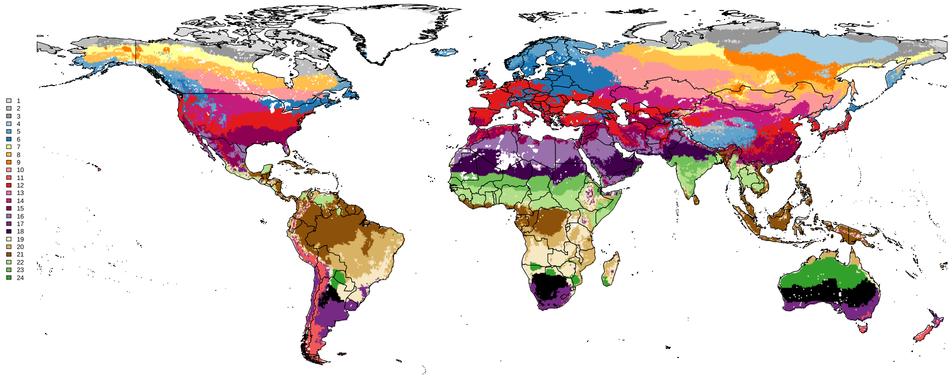

FIGURE S1 Environmental zones used in the stratified sampling algorithms (see methods). This map is the outcome of a classification of the input environmental data used by the species distribution models. The colours were selected to allow the viewer to distinguish zones and have no ecological interpretation.

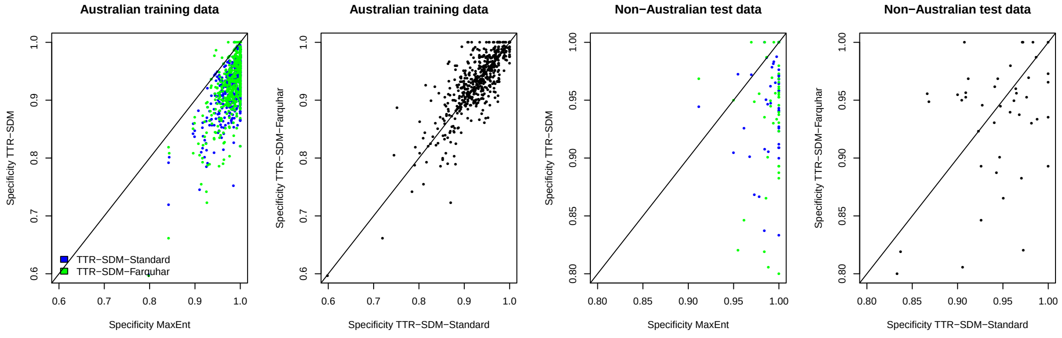

FIGURE S2 Pairwise comparison of the specificity of MaxEnt versus TTR-SDM models and for the standard version of the TTR-SDM versus the Farquhar version of the TTR-SDM. Australian training data indicates statistics calculated within the training region. Non-Australian test data indicates statistics for predictions made outside of Australia evaluated against GBIF records outside of Australia. Each data point represents a single species (n=664 for the training data, n=46 for the test data).

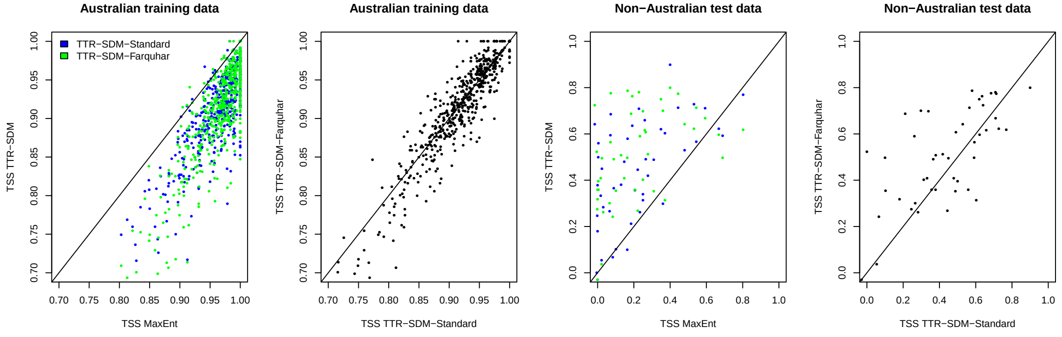

FIGURE S3 Pairwise comparison of the TSS statistics of MaxEnt versus TTR-SDM models and for the standard version of the TTR-SDM versus the Farquhar version of the TTR-SDM. Australian training data indicates statistics calculated within the training region. Non-Australian test data indicates statistics for predictions made outside of Australia evaluated against GBIF records outside of Australia. Each data point represents a single species (n=664 for the training data, n=46 for the test data).

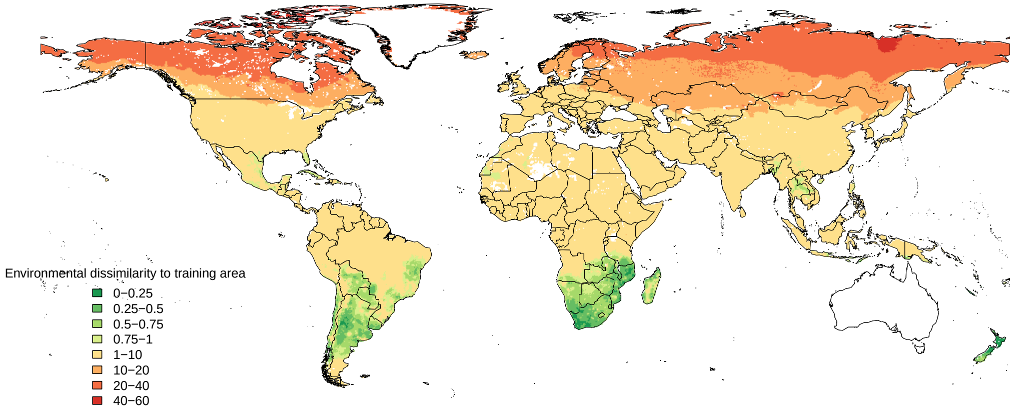

FIGURE S4 Environmental dissimilarity scores according to the $Nt2$ index (Mesgaran et al., 2014). The map indicates how dissimilar environmental variates are at locations outside of the testing arena (Australia). We used the trim.matrix function from the R package subselect (Orestes Cerdeira et al., 2020) to remove highly correlated variates prior to the calculation of the $Nt2$ scores.

references

Mesgaran, M. B., Cousens, R. D. and Webber, B. L. (2014) Here be dragons: a tool for quantifying novelty due to covariate range and correlation change when projecting species distribution models. *Diversity and Distributions*, **20**, 1147–1159. URL: `https://onlinelibrary.wiley.com/doi/abs/10.1111/ddi.12209`.

Orestes Cerdeira, J., Duarte Silva, P., Cadima, J. and Minhoto, M. (2020) *subselect: Selecting Variable Subsets*. URL: `https://CRAN.R-project.org/package=subselect`. R package version 0.15.2.
