# Supplementary material for: Predictive ability of a process‐based versus a correlative species distribution model
Source: Ecol Evol. 2020 Oct 8;10(20):11043–54. doi: 10.1002/ece3.6712 (PMC7593166; doi:10.1002/ece3.6712)
Supplement: Supplementary file 2 — Supplementary Material [file ECE3-10-11043-s002.pdf]

Supplementary Information for Predictive ability of a process-based versus a correlative species distribution model

Steven I. Higgins[1], Matthew Larcombe[2], Nicholas J. Beeton[3,4], Timo Conradi[1], and Henning Nottebrock[1]

[1]Plant Ecology, University of Bayreuth, Universitätsstraße 30, 95447 Bayreuth, Germany
[2]Department of Botany, University of Otago, PO Box 56, Dunedin 9054, New Zealand
[3]CSIRO, 3 Castray Esplanade, Battery Point, Tasmania 7004, Australia
[4]School of Biological Sciences, University of Tasmania, Private Bag 55, Hobart, Tasmania 7001, Australia

February 12, 2020

The Farquhar variant of the TTR-SDM uses user provided data on air temperature, atmospheric CO_2 concentration and photosynthetically active radiation to estimate a potential photosynthetic rate. The Farquhar variant of the TTR-SDM is exactly as described by Higgins et al. (2012), except that the photosynthesis function (see equation 14 in Higgins et al. 2012) is replaced by a Farquhar style photosynthesis function. Equation 14 in Higgins et al. (2012) states that the realised photosynthesis (A) is reduced from a potential maximal rate A_{max}, $A = A_{max} * \min(A_T, A_Q, A_W, A_N)$, where A_T, A_Q, A_W, A_N are functions that describe temperature, light, moisture and nitrogen limitations on A. The Farquhar variant of the TTR-SDM replaces this equation with $A = A_{fqr} * \min(A_W, A_N)$. Here A_{fqr} is the photosynthesis rate of the Farquhar model as influenced by temperature, light and atmospheric CO_2 concentration. The effects of moisture and nitrogen are included as before. This means that the parameters associated with this function (labelled $\beta_1 - \beta_8$ in Higgins et al. 2012) are no longer estimated from the distribution data, but are predefined by the Farquhar photosynthesis model (Farquhar et al., 1980). How we defined the C3 photosynthesis model is described in this supplementary information.

Our implementation of photosynthesis largely follows Von Caemmerer (2000). Since Von Caemmerer (2000) provides several model variants we write down the equations explicitly here. The equations and parameters are written in the form of R functions.

The model considers that the net leaf level photosynthesis in μmol.m^{-2}.s^{-1} is the minimum of a carbon fixation limited and a light-limited rate of CO_2 assimilation minus the leaf respiration. Each rate is influenced by temperature dependent enzymatic parameters. These temperature dependencies are either described by a non-peaked Arrhenius temperature function `npf` or by a peaked Arrhenius temperature `pf` function.

```
pf<-function(KTemp,E,S,H,y25,KTref=298,R=0.008314) { #peaked Arrhenius function
        firstexp<-exp(((KTemp-KTref)*E)/(KTref*R*KTemp))
        topexp<-(1+exp((KTref*S-H)/(KTref*R)))
        bottomexp<-(1+exp((KTemp*S-H)/(KTemp*R)))
        r<-y25*firstexp*(topexp/bottomexp)
        return(r)
}

npf<-function(KTemp,E,y25,KTref=298,R=0.008314) { #non-peaked Arrhenius function
    y25*exp((KTemp-KTref)*E/(KTref*R*KTemp))
}
```

The parameters used are stored in a list (`p3`). The comments in the list `p3` provide the source of the parameters and their units. We use units as in Von Caemmerer (2000). Parameter estimates are from Von Caemmerer (2000), Yin et al. (2011) and Patrick et al. (2009).

```
p3<-list()
    p3$Vcmax25   <- 80    # umol/m2/s von Caemmerer Table 2.3
    p3$ps2a25    <- 2     # unitless, see Yin
    p3$ps2b25    <- 2000  # unitless, see Yin
    p3$sprime    <-0.3115 # unitless, calibration factor see Yin

    p3$Kc25     <-260   # ubar #von Caemmerer Table 2.3
    p3$Ko25     <-179   # mbar #von Caemmerer Table 2.3
    p3$Gstar25  <-38.6  # ubar #von Caemmerer Table 2.3
    p3$gm       <-0.25  # mol/m2/s/bar # von Caemerer Figure 2.7
    p3$Rd25     <-0.01*p3$Vcmax25 # von Caemmerer Table 2.3

#--peak temp. dependences
    p3$EVcmax <-58.52   # kJ/mol   Patrick suppl. materials
    p3$SVcmax <-0.472   # J/mol/K  Patrick suppl. materials
    p3$HVcmax <-144.568 # kJ/mol Patrick suppl. materials

    p3$Eps2a <-74.  # kJ/mol    #  Yin
    p3$Sps2a <-0.4  #J/mol/K    #  Yin
    p3$Hps2a <-118. #kJ/mol     #  Yin

    p3$Eps2b <-87.  # kJ/mol     #  Yin
    p3$Sps2b <-0.39 # J/mol/K    #  Yin
    p3$Hps2b <-115. # kJ/mol     #  Yin

#--non-peak temp. dependences
    p3$EKc    <-59.36  # kJ/mol von Caemmerer Table 2.3
    p3$EKo    <-35.94  # kJ/mol von Caemmerer Table 2.3
    p3$EGstar <-27     # kJ/mol 23.4 von Caemmerer Table 2.3
    p3$Egm    <-49.6   # kJ/mol Patrick suppl. materials
    p3$ERd    <-66.4   # kJ/mol von Caemmerer Table 2.3

#--Ball-Berry stomatal conductance parameters
    p3$m<-9
    p3$b<-0.01
```

Atmospheric pressure, and the partial pressures of CO_2 and O_2 are assumed to be constant globally.

```
##----environmental constants / parameters
  ATM_P = 101.325*10^3    # Atmospheric pressure (Pa)
  ca<- (400/1e6)*ATM_P*10 # partial pressure co2 (bar)
  oa<- (21/100)*ATM_P*10  # partial pressure o2 (bar)
  oa<- oa/1000            # partial pressure o2 (mbar)
```

Leaf temperature `tl` (assumed to be the same as air temperature) and photosynthetically active radiation (`PAR`, μmol.m^{-2}.s^{-1}) are assumed to vary at each site.

The C3 model considers that the net leaf level photosynthesis in μmol.m^{-2}.s^{-1} is the minimum of the Rubisco-limited and the light-limited rate of rate of CO_2 assimilation minus the leaf respiration. Each rate is influenced by a temperature dependent enzymatic parameters. The Rubisco limited rate is

```
acC3 <- function(  cm, om, tl, pp ) {
   #--Gross Rubisco-limited photosynthetic rate (umol/(m^2s^1))

      vcmax<- pf(tl,pp$EVcmax,pp$SVcmax,pp$HVcmax,pp$Vcmax25)
        # Maximum carboxylation rate (umol/(m^2s))
      ko<-npf(tl,pp$EKo,pp$Ko25) # Michaelis-menten coefficient for O2
      kc<-npf(tl,pp$EKc,pp$Kc25) # Michaelis-menten coefficient for CO2
      Gstar<-npf(tl,pp$EGstar,pp$Gstar25) #Chloroplastic CO2 photocompensation point
      ac<-vcmax*(cm-Gstar) / ( cm + kc*(1 + om/ko ) )
      return(ac)
            }
```

There is less consensus on how to model the light dependency of photosynthesis. Most studies use an empirical sub-model such as a non-rectangular hyperbola (Von Caemmerer, 2000). We follow Yin et al. (2011) and explicitly model it as function of the photochemical quantum yield, also referred to as the quantum yield of Photosystem II (`ps2phi`). `ps2phi` can be directly estimated from measurements of chlorophyll fluorescence (e.g. Yin et al. 2011). We model that `ps2phi` has a dependency on the photosynthetically active radiation (`PAR`).

```
ajC3 <- function( cm, tl, PAR, pp) {
  #--Gross Electron transport - limited photosynthetic rate (umol/(m^2s^1))

    Gstar<-npf(tl,pp$EGstar,pp$Gstar25)  #Chloroplastic CO2 photocompensation point
    ps2a<- pf(tl,pp$Eps2a,pp$Sps2a,pp$Hps2a,pp$ps2a25)
    ps2b<- pf(tl,pp$Eps2b,pp$Sps2b,pp$Hps2b,pp$ps2b25)
    ps2phi <-  exp(-1/ps2a - 1/ps2b*PAR ) #dependecy of ps2phi on PAR
    # ps2phi: Electron flow through PS II per unit quantum flux (unitless)
    J <- pp$sprime*PAR*ps2phi #Electron transport rate umol/(m^2s^1)
    aj<- ( (cm-Gstar)*J ) / (4.5*cm + 10.5*Gstar)
    return(aj)
    }
```

The combined C3 function is then the minimum of minimum of the Rubisco-limited and the light-limited rate of rate of CO_2 assimilation minus the leaf respiration. This rate is influenced by the leaf temperature (`tl`) and the mesophyll CO_2 partial pressure `cm`.

```
aC3<-function( cm, om, tl, PAR, pp) {
  #--combined C3 function
    Rd<-npf(tl,pp$ERd,pp$Rd25)
    ac <- acC3(  cm, om, tl, pp )
    aj <- ajC3(  cm, tl, PAR, pp )
    return( min(ac,aj) - Rd )
  }
```

The photosynthesis equations are solved by finding the mesophyll CO_2 partial pressure `cm` that yields the same biochemical- `A` and diffusion- `Ad` based estimates of photosynthesis.

```
optC3<-function( cm, om,tl,PAR, p3, ca) {
    #--function to find the cm that minimizes abs(A-Ad)
        A<-aC3( cm, om, tl, PAR, p3 )
        h = 0.5 # relative humidity assumed constant at 50%
        gsc = (p3$m*a[i]*h/ca+p3$b)/1.6 # Ball-Berry stomatal conductance
        gm<-npf(tl,p3$Egm,p3$gm) # mesophyll conductance
        Ad<-(ca-cm) / (1/gsc+1/gm)
```

```
        d<-abs(A-Ad)
    }
```

References

Farquhar, G. D., von Caemmerer, S., and Berry, J. A. (1980). A biochemical model of photosynthetic CO2 assimilation in leaves of C3 species. *Planta*, 149(1):78–90.

Higgins, S. I., O'Hara, R. B., Bykova, O., Cramer, M. D., Chuine, I., Gerstner, E.-M., Hickler, T., Morin, X., Kearney, M. R., Midgley, G. F., and Scheiter, S. (2012). A physiological analogy of the niche for projecting the potential distribution of plants. *Journal of Biogeography*, 39(12):2132–2145.

Patrick, L. D., Ogle, K., and Tissue, D. T. (2009). A hierarchical bayesian approach for estimation of photosynthetic parameters of c3 plants. *Plant, Cell & Environment*, 32(12):1695–1709.

Von Caemmerer, S. (2000). *Biochemical Models of Leaf Photosynthesis*. Techniques in Plant sciences. CSIRO Publishing, Collingwood Australia.

Yin, X., Sun, Z., Struik, P. C., van der Putten, P. E. L., an Ieperen, W., and Harbinson, J. (2011). Using a biochemical c4 photosynthesis model and combined gas exchange and chlorophyll fluorescence measurements to estimate bundle-sheath conductance of maize leaves differing in age and nitrogen content. *Plant, Cell & Environment*, 34(12):2183–2199.
